# Drug Release Behavior of Enrofloxacin Molecularly Imprinted Polymer Synthesized via Electron Beam Irradiation

**DOI:** 10.3390/molecules31142447

**Published:** 2026-07-13

**Authors:** Xiang Li, Zixian Zheng, Jie Gao, Tao Chen

**Affiliations:** 1 School of Pharmacy, Hubei University of Science and Technology, Xianning 437100, China; danny_lxx@163.com (X.L.); 15039240974@163.com (Z.Z.); 2 Hubei Key Laboratory of Radiation Chemistry and Functional Materials, School of Nuclear Technology and Chemistry & Biology, Hubei University of Science and Technology, Xianning 437100, China

**Keywords:** imprinted polymer, electron beam irradiation, enrofloxacin, drug release

## Abstract

Traditional thermal polymerization for imprinted polymers suffers from long reaction time and initiator residues. This study proposes an initiator-free and fast reaction electron beam irradiation technique to synthesize enrofloxacin (ENRO) molecularly imprinted polymers (MIPs) for sustained drug release. ENRO-based MIPs were prepared using methacrylic acid (MAA) as a functional monomer and ethylene glycol dimethacrylate (EGDMA) as a crosslinker under electron beam irradiation within 2 h. The structure was characterized by SEM, FTIR, DSC, and TG. Drug loading capacity was determined via solvent elution, and drug release behavior was evaluated in simulated physiological environments. The MIP exhibited a high drug loading ratio of 13.27% and porous morphology (SEM). FTIR confirmed complete template removal, while thermal analysis indicated uniform drug dispersion and enhanced stability. Cumulative release was pH-dependent: 72.12% at pH 7.4 (physiological) and 41.67% at pH 1.2 (gastric). Drug release kinetics followed the Korsmeyer–Peppas model (R^2^ > 0.88), dominated by Fickian diffusion. Electron beam irradiation enables initiator-free synthesis of ENRO-based MIP with high loading capacity and pH-responsive sustained release, offering significant potential for targeted antibiotic delivery.

## 1. Introduction

Enrofloxacin (ENRO) is a third-generation broad-spectrum fluoroquinolone antibacterial agent with the molecular formula C_19_H_22_FN_3_O_3_. Its core structure comprises a fluorinated quinolone ring, a piperazinyl group, and a carboxylic acid moiety. The drug exerts potent bactericidal effects by inhibiting bacterial DNA gyrase, thereby disrupting DNA replication [1,2]. It demonstrates efficacy against a broad range of pathogens, including Gram-negative bacteria (e.g., *Pasteurella* spp., *Escherichia coli*), Gram-positive bacteria, as well as atypical organisms such as *Chlamydia*, *Mycoplasma*, and *Rickettsia*, showing significant activity against both stationary-phase and logarithmic-growth-phase bacteria. As the first veterinary-exclusive fluoroquinolone, ENRO is widely used in livestock for the treatment of bacterial diseases, demonstrating particular effectiveness in controlling colibacillosis, pasteurellosis, and *Haemophilus* infections. Its antibacterial activity is concentration-dependent, with bactericidal efficacy increasing proportionally with drug concentration [3,4,5].

To improve the drug loading efficiency, sustained release, and antibacterial efficacy of ENRO, various enrofloxacin drug delivery systems have been developed. To enhance the solubility and bioavailability of enrofloxacin, PVP-based oral thin films were developed, which demonstrated improved water uptake, favorable surface pH, uniform drug distribution, pH-dependent drug release, and dose-dependent antibacterial activity, particularly against *Klebsiella pneumoniae* [6]. ENRO-loaded PEG-silica capsules were developed and achieved a sustained release of up to 24 h with a drug loading of approximately 49.7%, and release kinetics were best described by the Sahlin–Peppas and Ritger–Peppas models [7]. γ-Cyclodextrin incorporated metal–organic framework (γ-CD-MOF) was fabricated for the delivery of ENRO, with a loading capacity of 45.25 mg/g, enabling sustained release over 4 h [8]. Beyond these, other sustained release systems for enrofloxacin such as nanoemulsions, liposomes, and mesoporous silica nanoparticles have also been explored [9]. Despite these advances, many of these systems are still associated with limitations including low loading capacity, complex preparation procedures, or insufficient long-term stability. Hence, there remains a continuing need to develop alternative platforms that combine high loading capacity, facile fabrication, and sustained release profiles [10].

Molecularly imprinted polymers (MIPs) possess three-dimensional memory cavities that enable on-loading and off-loading of various molecules [11], making them useful not only as a selective separation system [12,13] but also in a sustained delivery system for diverse drugs [14,15]. Carbamazepine (CBZ)-based MIPs were prepared using bio-based monomers such as eugenol and coumaric acid, achieving high adsorption (up to 50 mg/g) with a surface area of 437–560 m^2^/g, excellent selectivity for CBZ over diclofenac and ibuprofen, and stable recyclability over five adsorption–desorption cycles [16]. Rivastigmine tartrate (RVS)-based MIP nanoparticles were fabricated using methacrylic acid (MAA) and pentaerythritol triacrylate (PETA) at a molar ratio of 1:6:12 (RVS/MAA/PETA), achieving a high drug absorption of 77% and a sustained release over 168 h [17].

ENRO-based MIPs have also been widely reported. However, these studies predominantly focused on selective separation and detection [18,19], with very few investigations about the drug release of ENRO. Zeolitic-imidazolate-framework-67-supported surface MIPs were developed for ENRO selective separation, achieving a maximum adsorption capacity of 9.02 μg·mg^−1^, an imprinting factor of 2.58, rapid adsorption equilibrium within 30 min, and a detection limit as low as 0.23 ng·mL^−1^ with recoveries of 83.79–100.68% in real water samples [20]. A core–shell MIP-based potentiometric sensor was prepared for ENRO detection, achieving a linear response range of 1.0 × 10^−5^ to 1.0 × 10^−2^ M with a detection limit of 5 × 10^−6^ M and successful application in urine and soil samples with recoveries of 99.8–101.9% [21]. An electrochemical MIP sensor was developed for ENRO detection using Eichhornia-crassipes-derived nitrogen-doped carbon dots, achieving a wide linear range of 0.05–100 μM, an imprinting factor of 3.11, and satisfactory recoveries of 97.22–106.75% in lake water for ENRO detection [22]. In addition, all of the reported ENRO-based MIPs are prepared with initiator-induced thermal polymerization, suffering from problems of initiator residue and time consumption.

To address these drawbacks, electron beam (EB)-induced irradiation polymerization was applied for the preparation of MIPs, effectively eliminating initiator residues and shortening reaction times [23,24]. Nevertheless, to the best of our knowledge, the fabrication of ENRO-loaded MIPs via EB irradiation polymerization for sustained drug release has not yet been investigated. In this work, we therefore report the development of ENRO-based MIPs using EB irradiation technology. The resulting polymers were systematically characterized, and their drug release performance as well as release kinetics were thoroughly evaluated. This study does not merely introduce a new carrier but, more importantly, provides an initiator-free polymerization strategy that is particularly significant for veterinary drug formulations where safety and purity are paramount.

## 2. Results and Discussion

ENRO-based MIPs were synthesized via EB irradiation polymerization; then the chemical composition, morphology, and thermal properties were investigated, and the drug release behavior and release kinetics were revealed.

### 2.1. Characterization of ENRO-Based MIPs

#### 2.1.1. Chemical Composition Analysis

**Figure 1:**
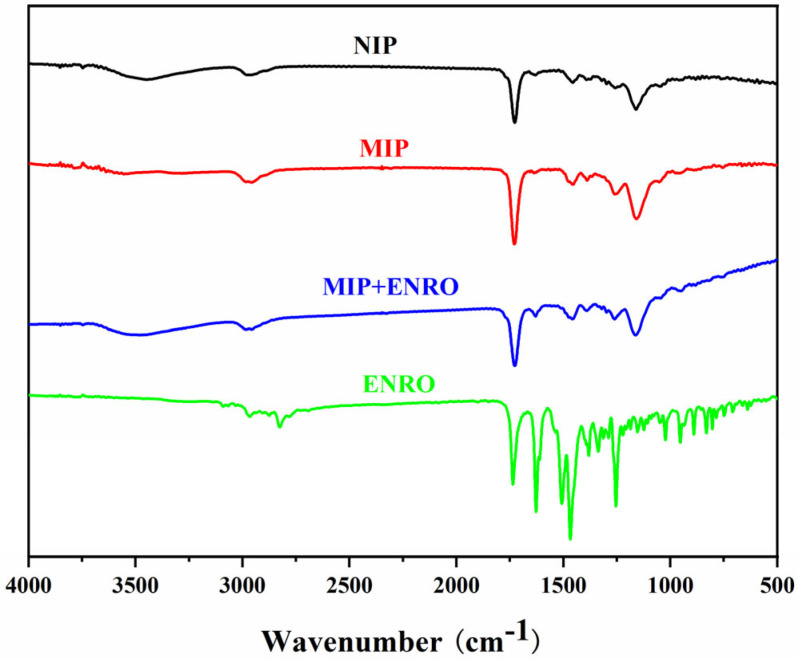
FTIR spectra of ENRO, MIP + ENRO, MIP, and NIP.

The chemical composition of ENRO, MIP + ENRO, MIP, and non-molecularly imprinted polymer (NIP) was revealed with the FTIR technique, as shown in Figure 1. For the FTIR spectrum of ENRO, the peaks at 1253, 1627, and 1738 cm^−1^ were assigned to C-F, C=N, and C=O stretching vibrations, and 1464, 2825, and 2962 corresponded to C-H vibration [6,8,9]. The characteristic peaks of C-F (1253 cm^−1^) and C=N (1627 cm^−1^) of ENRO have also appeared in MIP + ENRO. In addition, a broad peak (-OH, 3350~3650 cm^−1^) originated from methacrylic acid, and 2978 cm^−1^ was assigned to ethylene glycol dimethacrylate (-CH_2_-), which revealed the successful preparation of ENRO-loaded MIP [24]. For the FTIR spectrum of MIP, the peaks at 3350~3650, 2978, and 1735 cm^−1^ corresponded to -OH, -CH_2_-, and C=O vibration. The characteristic peaks of C-F and C=N of ENRO were not observed in the spectra of MIP, demonstrating the successful removal of ENRO template. Furthermore, the characteristic peaks of NIP are the same as those of MIP, which further revealed the complete removal of ENRO from MIP + ENRO.

#### 2.1.2. Morphology Analysis

**Figure 2:**
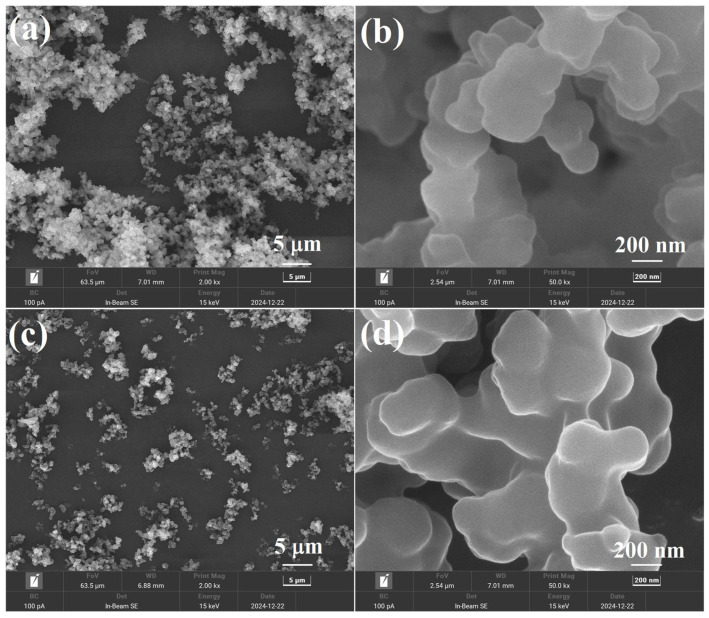
SEM images of MIP + ENRO (**a**,**b**) and MIP (**c**,**d**) with different sizes.

To further reveal the microstructure of the ENRO-based MIPs, the morphology of MIP + ENRO and MIP with different resolutions is presented in Figure 2. For MIP + ENRO, the low-magnification SEM image (Figure 2a) reveals irregular large particles with relatively smooth surfaces. At higher magnification (Figure 2b), the surface appears dense and exhibits few pores, as most of the pore channels are occupied and filled by ENRO molecules, resulting in restricted specific surface area and pore size. After the removal of the ENRO template molecules, the low-magnification image of the resulting MIP (Figure 2c) shows decreased particle sizes with slightly improved dispersity. At higher magnification (Figure 2d), the surface becomes distinctly porous, featuring an open and interconnected three-dimensional network of cavities with thinner pore walls. The observed morphological evolution corroborates the successful molecular imprinting and underscores the critical role of template elution in exposing functional recognition sites, thereby providing a structural foundation for subsequent release applications [20,24].

#### 2.1.3. Thermal Properties Analysis

**Figure 3:**
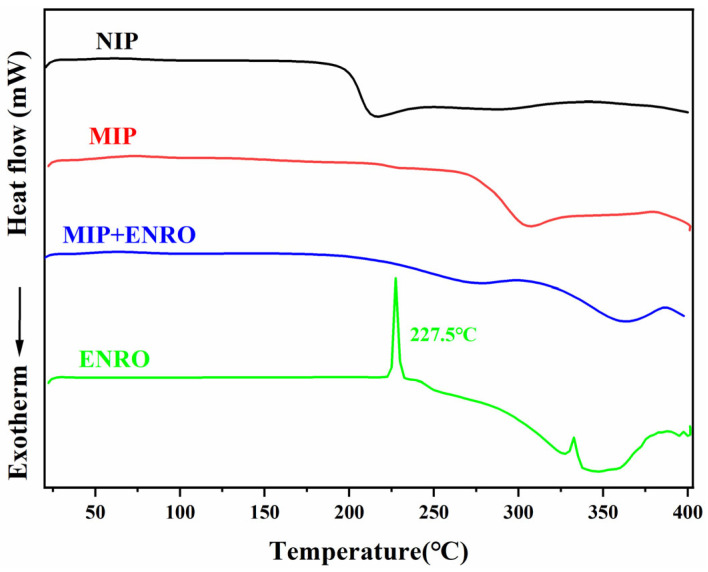
DSC curves of ENRO, MIP + ENRO, MIP and NIP.

The thermal effect of ENRO, MIP + ENRO, MIP, and NIP was determined with a differential scanning calorimeter (DSC), as shown in Figure 3. The pristine ENRO exhibits a sharp endothermic melting peak at 227.5 °C, corresponding to the disruption of its crystalline ordered structure, which indicates that enrofloxacin exists in a highly crystalline state [8]. For the template-loaded imprinted polymer MIP + ENRO, the melting peak completely disappears within the corresponding temperature range, confirming that ENRO is highly dispersed in the amorphous polymer matrix [25]. The intermolecular interactions are suppressed by the confinement effect of the imprinted cavities, thereby hindering crystallization behavior. Moreover, no obvious endothermic event is observed below 100 °C, suggesting the absence of low-boiling-point solvent residues. After the elution of ENRO, the resulting MIP exhibits an almost flat baseline throughout the entire heating range without any distinct thermal transition peaks. This indicates that the polymer backbone retains a rigid crosslinked structure with no glass transition process occurring within this temperature range, demonstrating excellent thermal stability, and that the cavity architecture remains intact without thermally induced collapse after template removal. The DSC curve of the non-imprinted polymer NIP is similarly flat and closely resembles that of MIP, and both are devoid of melting peaks, further confirming the absence of residual template molecules [26,27].

**Figure 4:**
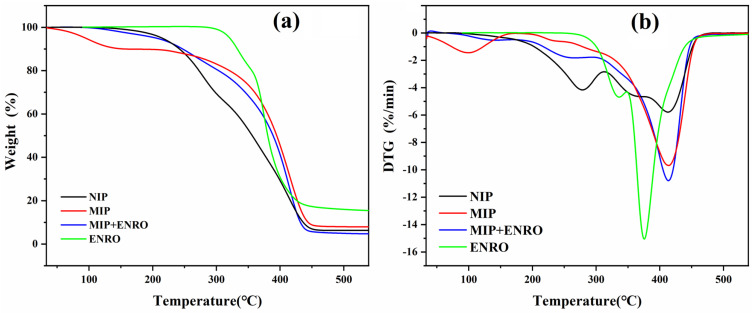
(**a**) TG and (**b**) DTG curves of ENRO, MIP + ENRO, MIP and NIP.

The thermal stability of ENRO, MIP + ENRO, MIP and NIP was investigated with a thermogravimetric analyzer (TG), as shown in Figure 4. As shown in Figure 4a, crystalline small-molecule ENRO exhibits a thermal weight loss of less than 1% at 300 °C, indicating its favorable thermal stability. Rapid decomposition occurs in the range of 300–450 °C, with a final residue mass of 17.5%. Compared with ENRO, both the thermal stability and the residual mass of the amorphous polymeric materials are significantly reduced [17,28]. MIP + ENRO and NIP display similar thermal decomposition behavior, with a weight loss of 6.3% below 230 °C. This early-stage weight loss could be primarily due to the volatilization of residual solvents or other low-molecular-weight species trapped during synthesis. Subsequently, gradual decomposition proceeds, and the residual mass remains nearly constant at approximately 6.6% after 450 °C. For MIP, the elution of ENRO exposes a substantial number of void structures, which might retain elution solvents or adsorb ambient moisture, resulting in a weight loss of up to 12.2% before 230 °C. This observation is consistent with the presence of accessible cavities in the MIP structure, although direct evidence of the evolved species would require further analysis. Thereafter, the decomposition behavior parallels that of NIP, with a final residual mass of 7.1%.

The maximum decomposition rates follow the order (Figure 4b): ENRO (~15.1%/min) > MIP + ENRO (10.8%/min) > MIP (9.7%/min) > NIP (5.8%/min). The temperature corresponding to the maximum decomposition rate of the small-molecule ENRO is 375.6 °C, whereas that of the three polymeric materials is approximately 412 °C. This difference might be explained by the synchronous cleavage of chemical bonds in small molecules, resulting in concentrated decomposition and a higher rate, whereas the hierarchical structures of polymeric materials would lead to a stepwise decomposition process and a higher temperature for the maximum decomposition rate [17,28]. However, we acknowledge that this interpretation remains speculative without direct structural or evolved-gas analysis.

### 2.2. In Vitro Release Behavior of ENRO

**Figure 5:**
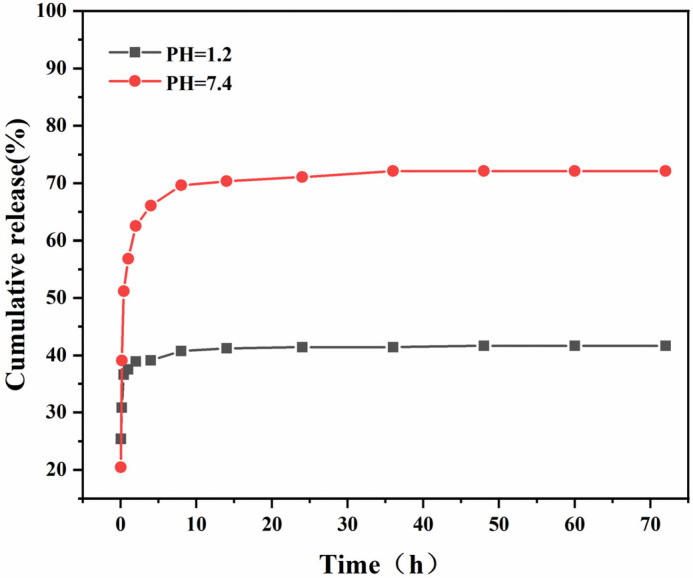
Cumulative release of ENRO under pH = 1.2 and pH = 7.4.

The drug release profiles of ENRO-based MIPs were evaluated in simulated physiological (pH 7.4) and gastric (pH 1.2) environments at 37 °C (Figure 5). As shown in Figure 5, ENRO-based MIPs exhibited pronounced pH-responsive release [27,28]. The cumulative release reached 72.12% over 72 h at pH = 7.4, which was attributed to deprotonation of MAA carboxyl groups (-COO^−^) weakening electrostatic attraction with ENRO’s piperazinyl moiety. The release at pH = 1.2 was suppressed to 41.67%, due to protonated MAA (-COOH) forming strong electrostatic bonds with ENRO and a shrunken polymer matrix restricting diffusion channels.

### 2.3. Release Kinetics Analysis

**Figure 6:**
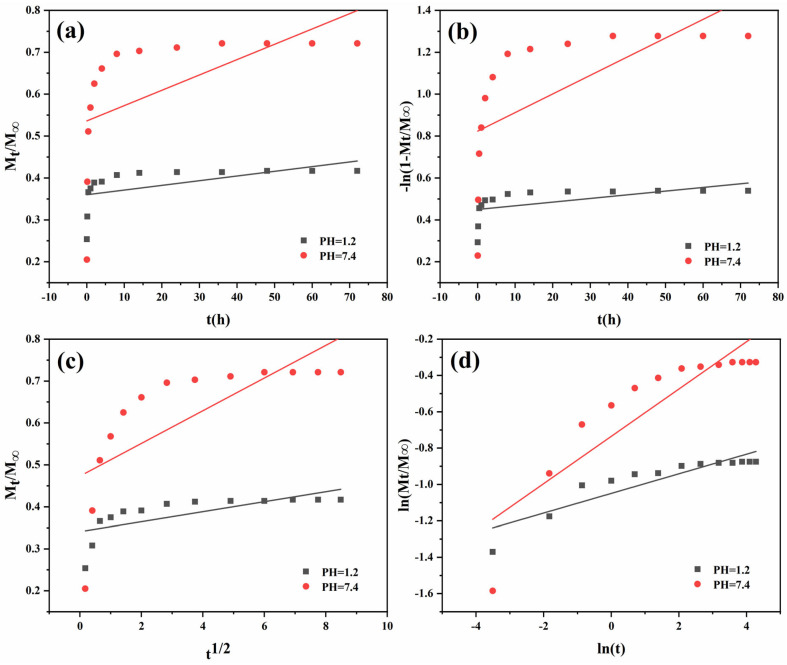
Linear fitted plots of the ENRO release with (**a**) Zero-order, (**b**) First-order, (**c**) Higuchi, and (**d**) Korsmeyer–Peppas models.

Four typical kinetic models, namely zero-order, first-order, Higuchi, and Korsmeyer–Peppas, were applied to evaluate the ENRO release performance from MIP + ENRO [9,25]. The linear fitted curves of ENRO release at different pH values are presented in Figure 6, and the kinetic parameters with corresponding coefficient (R^2^) calculated from these curves are listed in Table 1. As shown in the table, the Korsmeyer–Peppas model consistently yielded the highest R^2^ values (0.9069, 0.8893) for pH = 1.2 and 7.4, suggesting that drug release is primarily governed by diffusion [29,30,31]. The diffusion exponent *n* values were all below 0.5, indicating Fickian diffusion-controlled release rather than erosion-dominated release.

It is noteworthy that the remaining three models exhibited only moderate goodness of fit (R^2^ = 0.5689–07272), which warrants further mechanistic discussion. The poor performance of the zero-order model indicates that the drug release rate is not time-independent, which is expected for a reservoir-type system where the drug concentration gradient decreases over time [29]. The first-order model, which assumes release proportional to the remaining drug concentration, yielded unsatisfactory fits possibly because the heterogeneous binding sites within the imprinted cavities lead to non-uniform affinity, creating a more complex release profile than a simple first-order process [31]. The moderate fit of the Higuchi model, despite its relevance to diffusion-controlled release from porous matrices, suggests that the actual release deviates from ideal Higuchi assumptions. This deviation is likely due to the pH-responsive swelling of the MIP matrix, which may alter the diffusional path length over time [25].

### 2.4. Comparative Advantages of EB-Induced MIP Release System

On the one hand, compared with conventional thermal polymerization, electron beam (EB) irradiation can directly initiate free radical polymerization and crosslinking, thereby avoiding the residual toxicity of initiators such as AIBN and BPO [26,27]. Moreover, EB-induced polymerization significantly shortens the reaction time, typically completing within 1 h, which is far less than the 12–24 h required for conventional thermal polymerization [28,29,30,31]. Although gamma-rays have also been applied for the preparation of MIPs without an initiator, this approach typically requires approximately six hours of irradiation time. Additionally, gamma-ray irradiation relies on radioactive isotope sources such as ^60^Co, which necessitates specialized facilities and strict safety regulations, limiting its widespread applicability [32,33,34].

On the other hand, the prepared material exhibits a relatively high drug loading capacity of 13.27%, which is superior to that of γ-CD-MOF (4.5%) [10]. The drug release rates differ considerably at various pH values, indicating pH-responsive behavior [27,28]. Furthermore, the specific recognition of ENRO by the imprinted cavities lays a foundation for subsequent in vivo targeting studies [20].

## 3. Materials and Methods

### 3.1. Materials

Enrofloxacin (ENRO, 99%), methacrylic acid (MAA, AR), and ethylene glycol dimethacrylate (EGDMA, 99%) were provided by HWRK Chemical Co. Ltd. (Beijing, China). The MAA (containing 110 ppm MEHQ as inhibitor) was purified by vacuum distillation under reduced pressure to remove the MEHQ inhibitor, methanol (AR) and glacial acetic acid (AR) were purchased from Sinopharm Chemical Reagent Co., Ltd. (Shanghai, China). All other reagents were of AR grade and used without further purification. Deionized water was used throughout the experiments.

### 3.2. Preparation of ENRO-Based MIP

**Figure 7:**
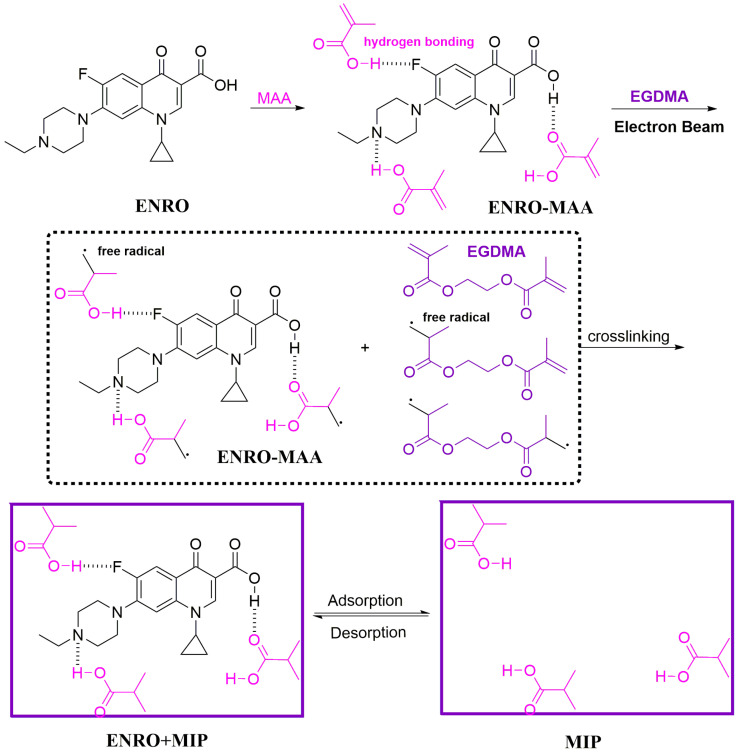
Preparation of ENRO-based MIPs via EB radiation polymerization.

The preparation of ENRO-based MIPs via EB irradiation polymerization is schematically illustrated in Figure 7 [24,35,36,37]. First, 4 mmol of ENRO (template molecule) and 16 mmol of MAA (functional monomer) were dissolved in 280 mL of methanol and magnetically stirred at 300 rpm for 2 h at 25 °C to allow the formation of ENRO-MAA complexes via hydrogen bonding between the carboxyl group of MAA and the keto/piperazinyl groups of ENRO. Subsequently, 80 mmol of EGDMA (crosslinker) was added to the solution, and nitrogen was bubbled through the mixture for 30 min to remove dissolved oxygen.

After sealing with PE bags, the mixture was irradiated using an electron accelerator (1 MeV, Wasik Associates, Haverhill, MA, USA) at a total dose of 400 kGy with a dose rate of 20 kGy/pass. Under electron beam irradiation, the vinyl groups of MAA and EGDMA undergo rapid free radical polymerization and crosslinking, forming a highly crosslinked three-dimensional polymer network. During this process, the pre-assembled ENRO-MAA complexes are immobilized within the network through the memory cavities created around the template molecules. The irradiation was carried out at ambient temperature without external heating, and the entire polymerization was completed within 2 h. All MIPs were prepared in triplicate (*n* = 3) to ensure batch-to-batch reproducibility.

Following irradiation, the resulting ENRO + MIP composites were washed with methanol to remove unreacted monomers and soluble oligomers. The purified composites were then subjected to Soxhlet extraction with a methanol/acetic acid (90/10, *v*/*v*) mixture to remove the template ENRO molecules. The extraction was continued until no ENRO was detectable in the eluent by UV–vis spectrophotometry at 320 nm. The final MIPs were dried under vacuum at 40 °C for 24 h.

**Figure 8:**
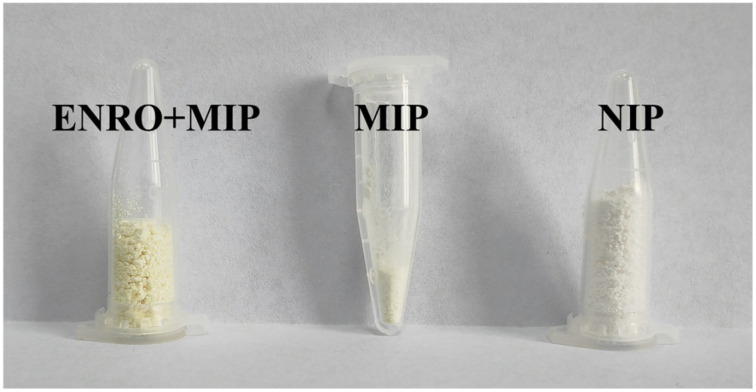
The pictures of ENRO + MIP, MIP, and NIP.

The non-imprinted polymer (NIP) was prepared following a procedure identical to that described above for the ENRO + MIP composites, with the sole exception that no ENRO was added during the pre-assembly step. Specifically, 16 mmol of MAA and 80 mmol of EGDMA were dissolved in 280 mL of methanol and subjected to the same stirring, nitrogen purging, and EB irradiation (1 MeV, 20 kGy/pass, 400 kGy cumulative dose). Finally, the obtained mixture was washed with methanol to remove unreacted reagents. The obtained NIP was dried under vacuum at 40 °C for 24 h. The picture of the obtained materials is shown in Figure 8.

### 3.3. Characterization

The prepared ENRO + MIP, MIP, and NIP were thoroughly characterized using a variety of techniques. The microscopic features and morphology were determined by scanning electron microscopy under 10 kV (SEM, ZEISS Gemini 300, Oberkochen, Germany). The composition of the obtained materials was revealed with Fourier transform infrared spectroscopy (FTIR, Avatar 360 Nicolet instrument, Thermo Fisher Scientific, Shanghai, China). Differential scanning calorimetry (DSC, DSC-200-F3, Netzsch, Selb, Germany) and a thermogravimeter (TG, TG-209-F3, Netzsch, Germany) were used in a nitrogen atmosphere. The absorbance of the ENRO solution was determined with UV–vis spectroscopy (UV-1000, Shanghai Aoyi, Shanghai, China) at 320 nm.

### 3.4. Drug Loading

The ENRO loading of ENRO + MIP was determined according to the reported method [8,25] and calculated with Equation (1). First, 400 mg of ENRO + MIP was weighed and placed into a mixture of 90 mL methanol and 10 mL glacial acetic acid and stirred for 8 h after 30 min of ultrasound treatment. After centrifugation at 8000 rpm for 15 min, the absorbance was measured, and the released amount of ENRO was calculated through Lambert’s law. The above procedure was repeated until no ENRO was detected in the eluent; the loading amount is the mass sum of ENRO eluted in each cycle. All drug loading determinations were performed in triplicate for each synthesized batch. The mass sum of ENRO is 53.08 ± 2.36 mg, and the drug loading is 13.27 ± 0.59%.

Drug Loading (%) = (Determined drug loading/total weight) × 100 (1)

The standard concentrations of ENRO ranged from 1 to 10 mg/L (1.0, 2.0, 4.0, 6.0, 8.0, 10.0 mg/L) for drug loading determination. The linear regression equation is: *A* = 0.0392 *C* − 0.0039 (R^2^ = 0.9973), where *A* is the absorbance and *C* is the concentration in mg/L.

### 3.5. In Vitro Release of ENRO

The release of ENRO was investigated under pH = 1.2 and 7.4 at 37 °C [9,10,25]. Typically, 50 mg MIP + ENRO was placed in 100 mL buffer solution. Sustained release liquid was collected at a specific time and measured with a UV–vis spectrophotometer (UV-1000, Shanghai Aoyi, Shanghai, China); then an identical amount of new buffer was added. The concentration of ENRO in the collected samples was determined by UV–vis spectrophotometry at 320 nm. All release experiments were conducted as single tests (*n* = 1). Cumulative drug release percentage *Q_t_* was calculated according to the following Equation (2):

*Q_t_* = [(*C_t_* × *V*_0_ + *V* × ∑*C_t_*_−1_)/*m*] × 100% (2)

where *m* is the weight of the loaded drug, *C_t_* is the drug concentration at the moment t, *C_t−_*_1_ is the concentration at the moment t – 1, *V*_0_ is the total volume, and *V* is the volume of the sample.

The standard concentrations of ENRO ranged from 1 to 10 mg/L (1.0, 2.0, 4.0, 6.0, 8.0, 10.0 mg/L) for cumulative drug release determination. The linear regression equations are *A* = 0.0491 *C* + 0.0060 (R^2^ = 0.9958) and *A* = 0.0318 *C* + 0.0121 (R^2^ = 0.9965) for pH = 1.2 and 7.4., respectively.

### 3.6. Release Kinetics of ENRO

The release kinetics of ENRO from MIP were fitted with four kinetic models, namely zero-order, first-order, Higuchi, and Korsmeyer–Peppas models (Equations (3)–(6)) [8,9,10].

*M_t_*/*M_∞_* = *K*_0_
*t* (3)

ln(1 − *M_t_*/*M_∞_*) = −*K*_1_*t* (4)

*M_t_*/*M_∞_* = *K_H_t*^1/2^ (5)

*M*_*t*_/*M*_∞_ = *K*_*KP*_*t*^*n*^ (6)

where *M_t_* and *M_∞_* are the mass of drug dissolved at time t and after infinite time. K_0_ is the kinetic dissolution constant, K_1_ and K_H_ are the kinetic constants, K_KP_ is the proportional constant, and *n* is the diffusional exponent.

## 4. Conclusions

In summary, ENRO-based MIPs were successfully synthesized via initiator-free electron beam irradiation technology. This approach overcame two major drawbacks of conventional thermal polymerization, namely prolonged reaction time and initiator residue. Structural characterization confirmed the formation of imprinted cavities. SEM revealed a porous morphology after template elution. The disappearance of the C-F vibration peak (1253 cm^−1^) in FTIR spectra confirmed complete removal of the template molecules. Thermal analysis (DSC/TG) demonstrated uniform dispersion of ENRO in an amorphous state and enhanced thermal stability of the MIPs. The resultant MIPs exhibited excellent drug loading performance (loading capacity of 13.27%) and pH-responsive release characteristics. The cumulative release rate reached 72.12% over 72 h in a simulated physiological environment (pH 7.4), whereas it decreased to 41.67% in a gastric acid environment (pH 1.2). This is attributed to the electrostatic interactions modulated by the protonation state switching of MAA carboxyl groups. The release kinetics conformed to the Korsmeyer–Peppas model (R^2^ > 0.88), with a diffusion exponent *n* < 0.45, confirming a Fickian diffusion mechanism. This work provides an initiator-free and efficient platform for antibiotic delivery. To further translate this system toward practical applications, our ongoing research will focus on evaluating the antibacterial activity of ENRO-loaded MIPs and exploring scalable continuous preparation methods for potential industrial production. These investigations will determine the feasibility of this MIP-based delivery system for veterinary clinical applications.

## Figures and Tables

**Table 1:** Kinetic parameters of four models for ENRO release.

Kinetic Model	Parameters	pH = 1.2	pH = 7.4
Zero-order	K_0_ (h^−1^)	0.0011	0.0037
	R^2^	0.5689	0.5822
First-order	K_1_ (h^−1^)	0.0018	0.0089
	R^2^	0.5833	0.6552
Higuchi	K_H_ (h^−1/2^)	0.0119	0.0390
	R^2^	0.7087	0.7272
Korsmeyer–Peppas	*n*	0.0540	0.1302
	K_KP_ (h^−*n*^)	0.3503	0.4794
	R^2^	0.9069	0.8893

## Data Availability

The original contributions presented in this study are included in the article. Further inquiries can be directed to the corresponding authors.
